# Biomechanical evaluation of the influence of posterolateral corner structures on cruciate ligaments forces during simulated gait and squatting

**DOI:** 10.1371/journal.pone.0214496

**Published:** 2019-04-04

**Authors:** Kyoung-Tak Kang, Yong-Gon Koh, Ji-Hoon Nam, Moonki Jung, Sung-Jae Kim, Sung-Hwan Kim

**Affiliations:** 1 Department of Mechanical Engineering, Yonsei University, Seoul, Republic of Korea; 2 Joint Reconstruction Center, Department of Orthopaedic Surgery, Yonsei Sarang Hospital, Seoul, Republic of Korea; 3 AnyBody Technology A/S, Aalborg, Denmark; 4 Department of Orthopedic Surgery, Arthroscopy and Joint Research Institute, Yonsei University College of Medicine, Gangnam Severance Hospital, Seoul, Republic of Korea; University of Rome, ITALY

## Abstract

Posterolateral corner (PLC) structures of the knee joint comprise complex anatomical soft tissues that support static and dynamic functional movements of the knee. Most previous studies analyzed posterolateral stability *in vitro* under static loading conditions. This study aimed to evaluate the contributions of the lateral (fibular) collateral ligament (LCL), popliteofibular ligament (PFL), and popliteus tendon (PT) to cruciate ligament forces under simulated dynamic loading conditions by using selective individual resection. We combined medical imaging and motion capture of healthy subjects (four males and one female) to develop subject-specific knee models that simulated the 12 degrees of freedom of tibiofemoral and patellofemoral joint behaviors. These computational models were validated by comparing electromyographic (EMG) data with muscle activation data and were based on previous experimental studies. A rigid multi-body dynamics simulation using a lower extremity musculoskeletal model was performed to incorporate intact and selective resection of ligaments, based on a novel force-dependent kinematics method, during gait (walking) and squatting. Deficiency of the PLC structures resulted in increased loading on the posterior cruciate ligament and anterior cruciate ligament. Among PLC structures, the PT is the most influential on cruciate ligament forces under dynamic loading conditions.

## Introduction

Isolated injuries of the posterolateral corner (PLC) structures of the knee are generally uncommon and can be easily overlooked in knee joint inspections, especially when there are concomitant with anterior cruciate ligament (ACL) and posterior cruciate ligament (PCL) tears [1–4]. The PLC structures of the knee have recently come to the fore as their significance has been supported by detailed anatomical descriptions, biomechanical researches and kinematic studies on their interactions with ACL and PCL [5–7].

In the past decade, the complexity of the PLC structures in the knee joint has been revealed, biomechanically and clinically [8, 9]. The key elements of the PLC of the knee are the lateral collateral ligament (LCL), popliteofibular ligament (PFL), and popliteus tendon (PT). Of these, the LCL and PFL are considered the primary static stabilizers of the PLC structures. The PT is a dynamic stabilizer of the knee, because the tendon covers the region from the PLC of the proximal part of the tibia to the musculotendinous junction. The PLC structures and the PCL work together to resist external tibial rotation and posterior tibial translation. In addition, the PLC structures that have been reported to be contributory to most joint movements have different effects, depending on static and dynamic loading conditions. These structures are functionally loaded in several joint loading conditions, and they could function either as primary or important secondary stabilizers during joint motion testing [10, 11]. Using a selective resection technique, Gollehon et al. evaluated the structures of the PLC that have the greatest influence on posterolateral instability [10]. Although the selective sectioning method of assessing the importance of specific, or a group of, structures for providing static stability to the knee is useful, cutting these structures changes the intricate interactions and relationships between the remaining knee structures and cancels the effect of the sectioned structure. Therefore, the results yielded by this method depends on the sequence in which the structures are resected [12, 13]. A number of in vitro studies have reported the effects of resecting the PLC structures on PCL force and posterolateral stability under static loading conditions, (e.g., laboratory experiments using cadaver legs under physiological static conditions) [12–14]. However, no in vitro study has analyzed posterolateral stability under dynamic conditions.

Finite element (FE) models are advantageous for estimating internal tissue stress and strains under dynamic conditions [15]; however, it is computationally challenging to use FE models for multi-body dynamic simulations of gait and squatting [16]. Recently, several musculoskeletal (MSK) multi-body dynamic models involving a deformable joint contact model have been developed [17–19]. Multibody dynamic MSK models offer a practical alternative for performing dynamic simulations, in which elastic ligament bundles capture the overall kinematic behavior of the joint [20–22].

The objective of this study was to develop and validate a subject-specific MSK lower extremity model for five subjects (four males and one female) that allowed a 12-degrees of freedom (DOF) of motion at the tibiofemoral (TF) and patellofemoral (PF) joints. First, muscle activation obtained using the subject-specific MSK models were compared with the transformed electromyography (EMG) measurements under gait- and squat-loading conditions for validation. Second, external rotation test results for an intact and deficient PLC structures conditions were compared with previous published data of static cadaver experimental results [37]. Finally, to evaluate the contribution of the PLC structures on cruciate ligaments force, PLC structure resection was performed virtually and investigated under gait and squatting loading conditions. We hypothesized that, of all PLC structures, PT is the most dominant dynamic stabilizer of the PCL force under dynamic loading conditions and that deficient PLC structures would lead to increased PCL force.

## Materials and methods

### Experimental measurements

Five healthy volunteers (four males and one female) participated in this study after providing written informed consent; The study protocol was approved by our institutional review board (Gangnam Severance Hospital, Yonsei University College of Medicine, Republic of Korea, IRB #3-2016-0083). All subjects participating in this study were volunteers, and no one dropped out. They had no previous medical history of lower extremity problems. The mean age, height, and weight of the subjects were 33.0 ± 4.4 years, 175 ± 7.4 cm, and 75.6 ± 6.7 kg, respectively.

**Fig 1:**
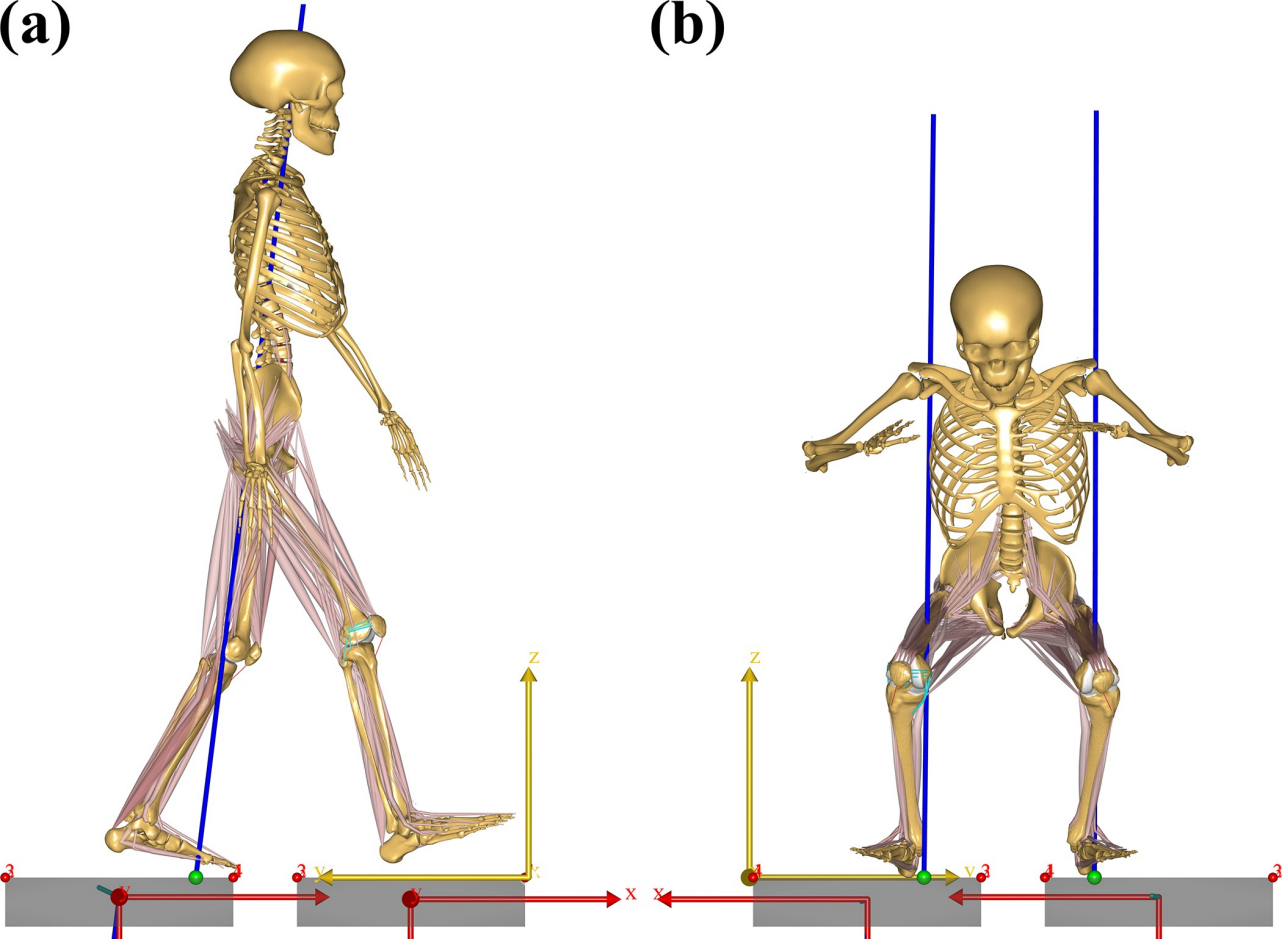
Subject-specific musculoskeletal models under the (a) gait and (b) squat loading conditions.

Subjects performed four trials of gait (walking) and squatting activities, and ground reaction forces were measured using a force plate (Fig 1). In addition, tracks of marker locations were measured using a three-dimensional (3D) motion-capture system (Vicon, Oxford, UK). EMG signals were recorded by an EMG sensor (Delsys, Boston, MA, USA) for the gluteus maximus, rectus femoris, vastus lateralis, biceps femoris, semimembranosus, gastrocnemius medialis, tibialis anterior, and soleus medialis.

To evaluate the predicted muscle activations of the MSK model, an EMG-to-activation model was developed to represent the underlying muscle activation dynamics. The process of transforming EMG data to muscle activation data has been reported previously [23].

### Subject-specific musculoskeletal models

Subject-specific models were developed using the AnyBody Modeling System (AMS, version 6.0.5; AnyBody Technology, Aalborg, Denmark), are for to analyze MSK simulation. The generic lower extremity MSK models were based on the Twente Lower Extremity Model (TLEM) anthropometric database [24]. The MSK model was actuated by approximately 160 muscle units, its ability to predict muscle and joint reaction forces in human lower limbs during locomotion was validated previously [25, 26].

The 3D bone and soft tissue models were reconstructed from computed tomography (CT) and magnetic resonance imaging (MRI) images from previous studies [27, 28]. Ligament insertion points were also obtained from the MRI images. The generic femur and tibia models of the TLEM in the AnyBody model were scaled to the 3D models of the femur and tibia of the subjects using non-linear radial basis functions that served as scaling laws. [29] The remaining segments were scaled using an optimization scheme that minimized the differences between model markers and recorded marker positions during dynamic conditions. The knee joint in this study consisted of 12 DOF (TF: 6 DOF, PF: 6 DOF). The hip and ankle joints were modeled as 3 DOF- and 2-DOF, respectively.

**Fig 2:**
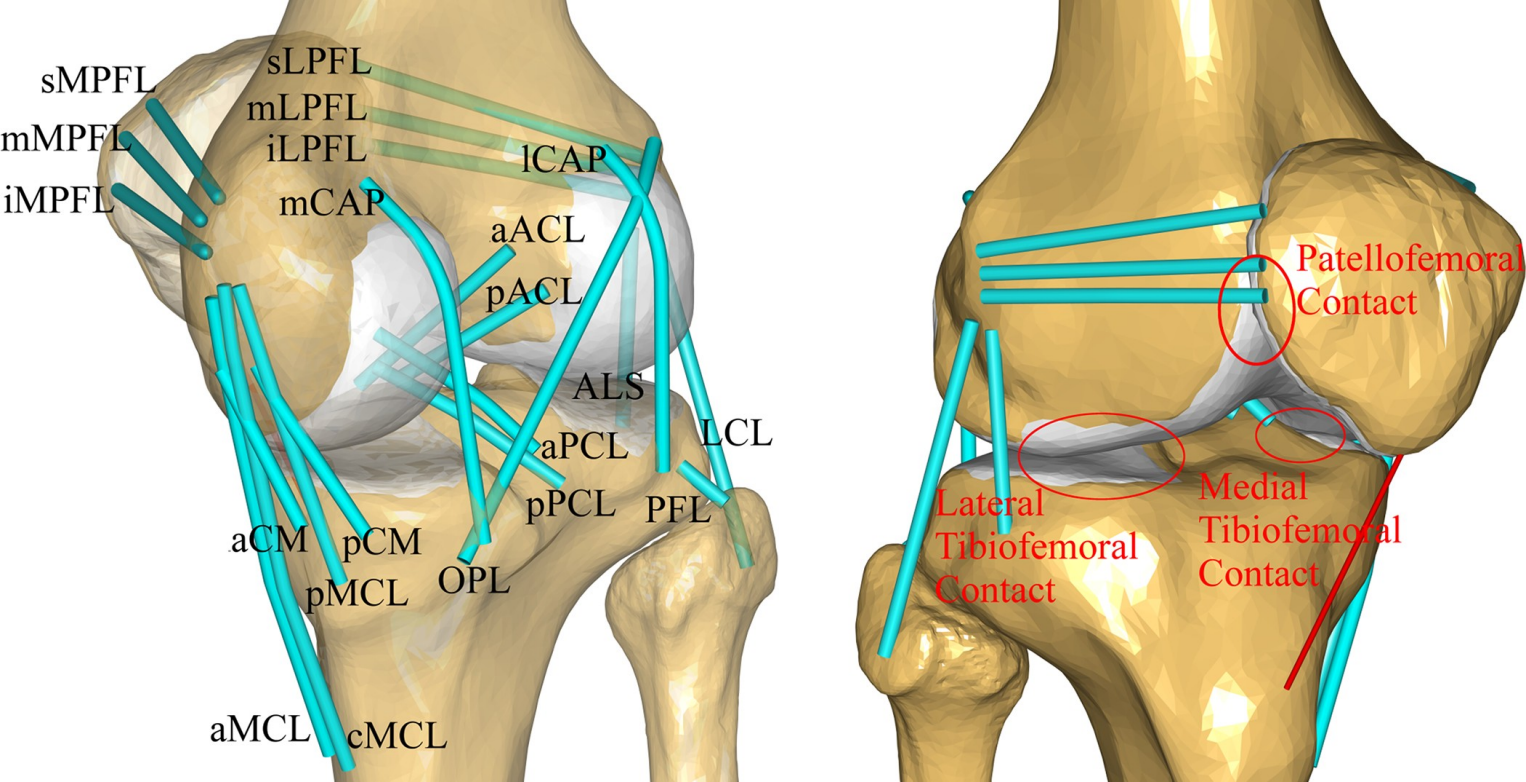
Schematic of the knee model with contact conditions and 21 ligament bundle: The anterior cruciate ligament (aACL, pACL) (anterior, a; posterior, p), posterior cruciate ligament (aPCL, pPCL), anterolateral structures (ALS), lateral collateral ligament (LCL), popliteofibular ligament (PFL), medial collateral ligament (aMCL, cMCL, pMCL) (a, anterior; c, central; p, posterior), deep medial collateral ligament (aCM, pCM), posterior capsule (mCAP and lCAP) (m, medial; l, lateral), oblique popliteal ligament (OPL), medial PF ligament (sMPFL, mMPFL, iMPFL) (s, superior; m, middle; i, interior), and lateral PF ligament (sLPFL, mLPFL, iLPFL).

The ligament attachment sites were obtained from the subject’s MRI results and from descriptions that can be found in the literature [30–32]. Two experienced orthopedic surgeons independently determined the locations of the ligaments. Agreement was evaluated using the 3D coordinates of each point. Intraclass correlation coefficients for intrarater- and interrater agreement ranged from 0.86 to 0.96 for all measurements, thus indicating good reproducibility [33]. The attachment points in the AnyBody model were modified using the subject-specific attachment sites. As shown in Fig 2, the following ligament bundles were modeled: the anterior cruciate ligament (aACL, pACL) (anterior, a; posterior, p), posterior cruciate ligament (aPCL, pPCL), anterolateral structures (ALS), lateral collateral ligament (LCL), popliteofibular ligament (PFL), medial collateral ligament (aMCL, cMCL, pMCL) (a, anterior; c, central; p, posterior), deep medial collateral ligament (aCM, pCM), posterior capsule (mCAP and lCAP) (m, medial; l, lateral), oblique popliteal ligament (OPL), medial PF ligament (sMPFL, mMPFL, iMPFL) (s, superior; m, middle; i, interior), and lateral PF ligament (sLPFL, mLPFL, iLPFL). The force-elongation relationship of the ligaments in this model was defined in order to produce a nonlinear elastic characteristic with a region of slack [34]:

$$f(\varepsilon )=\left\{ \begin{array}{l} \frac{k\varepsilon^{2}}{4\varepsilon_{1}},\;0\leq \varepsilon \leq {2\varepsilon }_{1}\\ k(\varepsilon -\varepsilon_{1}),\;\varepsilon >2\varepsilon_{1}\\ 0,\;\varepsilon <0 \end{array}\right.$$

$$\varepsilon =\frac{l-l_{0}}{l_{0}}$$

$$l_{0}=\frac{l_{r}}{\varepsilon_{r}+1}$$

where *f*(ε) is the current force, *k* is the stiffness, *ε* is the strain, and *ε*_*1*_ is assumed to be constant at 0.03. The slack length of the ligament bundle, *l*_*0*_, can be calculated by the reference bundle length, *l*_*r*_, and the reference strain, *ε*_*r*_, in the upright reference position.

Most of the stiffness and reference strain values were adopted from the literature and some were modified [20, 29, 34]. The material properties used in this literature are listed in Table 1. The menisci were modeled as linear springs to simulate their equivalent resistance [35]. Wrapping surfaces (cylindrical and ellipsoid) were applied to prevent the penetration of ligaments into bones. Between one the three wrapping surfaces were applied to each ligament to illustrate the geometry of the bone.

**Table 1:** Material properties in the ligaments used in this study.

	Stiffness (N)	Reference strain	Slack length (mm)
aACL	5,000	0.06	33.74
pACL	5,000	0.10	28.47
aPCL	9,000	-0.10	33.81
pPCL	9,000	-0.03	34.92
LCL	4,000	0.06	57.97
aMCL	2,500	-0.02	86.54
cMCL	3,000	0.04	84.72
pMCL	2,500	0.05	51.10
PFL	4,000	0.06	43.54
OPL	2,000	0.07	80.21
mCAP	2,500	0.08	60.13
lCAP	2,500	0.06	55.59
ALS	2,000	0.06	31.69
aCM	2,000	-0.27	37.53
pCM	4,500	-0.06	34.48
sMPFL	2000	0.10	59.58
mMPFL	2000	0.10	59.17
iMPFL	2000	0.10	59.41
sLPFL	1000	0.15	56.41
mLPFL	1000	0.15	56.21
iLPFL	1000	0.15	53.85

Fig 2 shows three rigid STL-based contacts defined in the TF and PF joints. These contact forces were proportional to the penetration volume and so-called pressure module [29]. The equation derived by Fregly et al was used to calculate the value of the pressure module. The pressure module value was 9.3 x 109 N/m3[17, 36].

### Popliteus muscle modification

**Fig 3:**
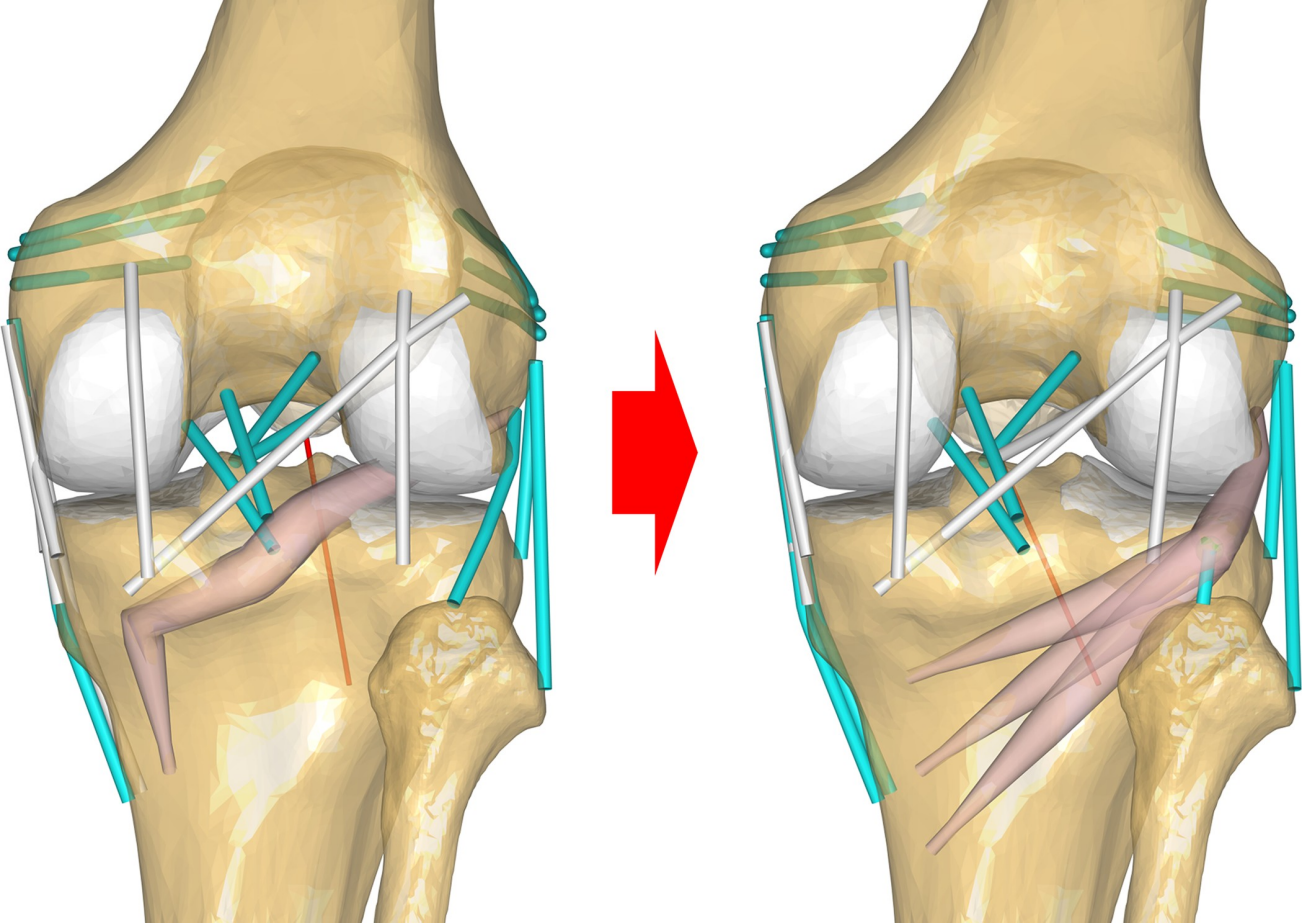
Schematic of the popliteus muscle modification.

The PT in the PLC structures provided by AnyBody was modified because it did not appear realistic (Fig 3) [8, 9, 13, 14]. The default popliteus muscle, which was composed of two bundles, was modified to have a third bundle. The tibia origin was modified so that it was located on each different anatomical site obtained using MRI. PFL was modified as to be connected with the popliteus muscle. The PFL that originates at the popliteus musculotendinous junction and attaches on the medial downslope of the fibular styloid process. [37]

### Inverse dynamic simulation and loading conditions

Gait and squat kinematics were calculated based on motion-capture data and using kinematic optimization. To optimize the kinematic model parameters of each joint which are displacements, velocities and accelerations, ground reaction forces and motion-capture-marker trajectory data were imported into AnyBody software. The objective of optimization was to minimize the difference between the AnyBody model marker trajectories and the motion-capture marker trajectories. After kinematic optimization, a force-dependent kinematics analysis was performed. In this study, a cubic polynomial was the muscle recruitment criterion. [29, 38]

To validate the MSK model, muscle activations were calculated by an inverse dynamic analysis, and compared with the EMG signals during the gait loading and squat loading conditions. The data were normalized before comparisons. For additional validation, external rotation torque tests to determine rotational laxity and 5 Nm of torque at 0°, 30°, 60°, and 90° of flexion of intact and deficient PLC structures were performed, and the results were compared with previously published data obtained from an experimental static cadaver study [39].

To define the influence of resection of the PLC structures on cruciate ligament forces, the forces with deficiencies in individual components (i.e., LCL, PFL, and PT) and, PLC structures as a whole (i.e., entire PLC structures), were investigated under gait loading and squat loading conditions.

### Statistical analysis

Cycles of gait and squatting were divided into 11 time points (0.0 to 1.0 phases). To assess 4 deficient conditions LCL deficient, PFL deficient, PT deficient, and entire PLC deficient each deficient condition was compared to the intact condition in pairwise manner made using non-parametric repeated-measure Friedman tests at each phase of the cycle. The post-hoc comparisons were performed using a Wilcoxon's rank test with Holm correction to control the familywise error rate for the tests conducted within each phase of the cycle. Statistical analyses were performed using SPSS for Windows (version 20.0.0; SPSS Inc., Chicago, IL, USA). Statistical significance was set at P < .05 for all comparisons.

## Results

### Comparisons between experimental EMG and muscle activation simulation measurements

The greatest muscle activities predicted by the five computational models showed consistency with the transformed EMG measurements under the gait loading and squat loading conditions (S1 Fig).

### Comparisons of external rotation between the simulation and an experimental cadaver study

**Fig 4:**
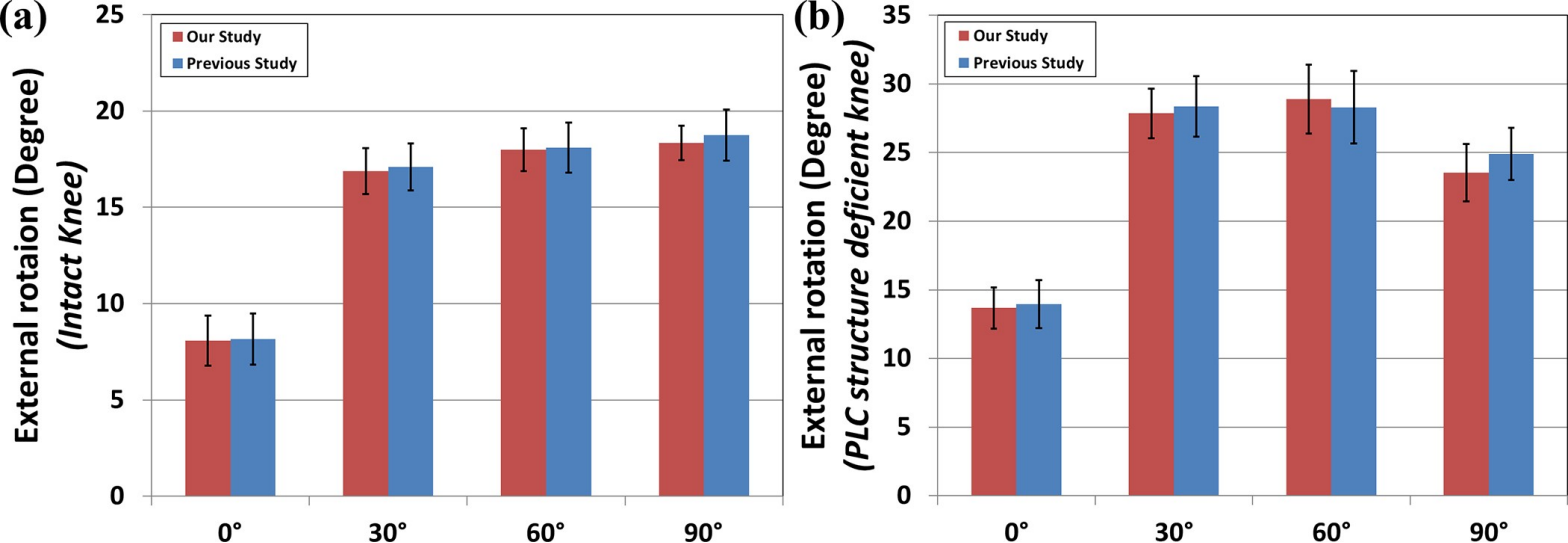
Comparison of the external rotation in the external rotation torque for experiment and computational simulation between the (a) intact and (b) PLC deficient conditions.

For additional validation of the external rotation simulations, the external tibial rotation value of the intact knee (8.09°, 16.88°, 17.99°, and 18.34°, at 0°, 30°, 60°, and 90° of flexion, respectively) and, those of the deficient knee of the PLC structure (13.67°, 27.86°, 28.89°, and 23.54° at each of the respective knee flexions) were determined. For the intact and PLC deficiency models, the mean values of simulated internal rotation were within the range of values of a previous experiment (Fig 4) [39].

### Cruciate ligament force under gait and squat loading conditions

**Fig 5:**
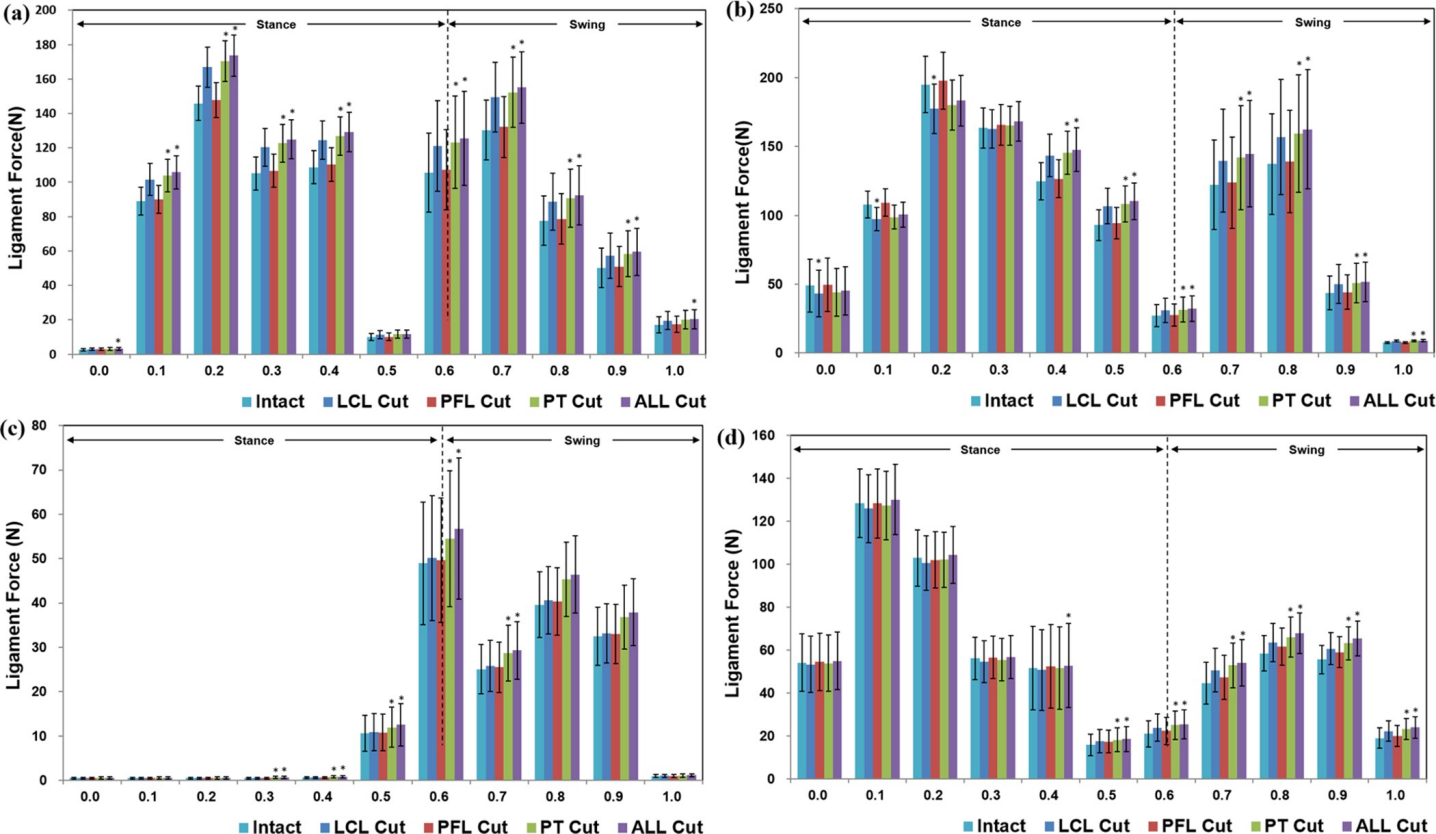
Mean (±SE) force exerted on the (a) aACL, (b) pACL, (c) aPCL, and (d) pPCL with deficiencies of the LCL, PFL, PT, and PLC structures under the gait loading condition (*P < .05).

Fig 5 shows forces in the cruciate ligaments with deficiencies of the LCL, PFL, and PT, as well as the entire PLC structure, under gait conditions. FDeficiency of the PT and the entire PLC structures also significantly influenced the forces on aACL and pACL. The deficiency of LCL and PFL significantly influenced the force on pACL during the early stance phase but did not influence aACL, aPCL and pPCL. In addition, during gait-loading conditions, ACL was more influenced than PCL by the deficiency of PLC structures.

**Fig 6:**
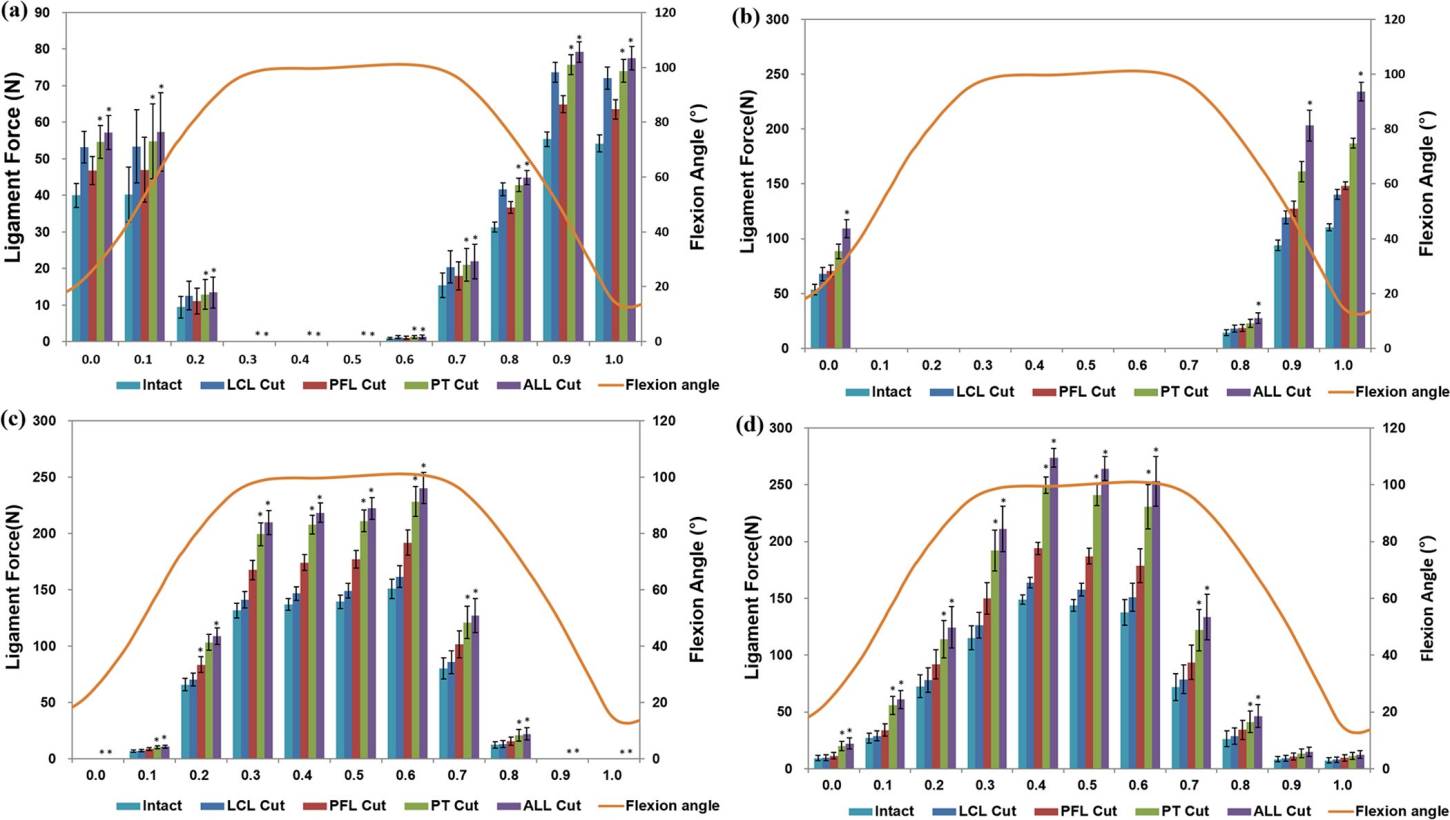
Mean (±SE) force exerted on the (a) aACL, (b) pACL, (c) aPCL, and (d) pPCL with deficiencies of the LCL, PFL, PT, and PLC structures under the squat loading condition (*P < .05).

Fig 6 shows the forces on the cruciate ligaments with deficiencies of the PT, PFL, LCL, and the entire PLC structure during squatting. As expected, the cruciate ligament force increased as the PLC structures were sectioned during the squat-loading conditions. However, unlike during gait-loading conditions, the rate of increase in the load markedly increased in the PCL during the squat-loading conditions compared to the ACL when the PLC structures were sectioned. The deficiency of the PT and all PLC structures also significantly influenced the force on the ACL. However, the force on the PCL was significantly influenced during the squat loading conditions. Similar to occurerences during the gait cycle, deficiency of the LCL and PFL did not significantly influence the forces on the ACL and PCL.

## Discussion

The most important finding of this study was that the deficiency of PLC structures significantly affected not only the PCL but also the ACL, which was more frequently shown under gait cycle conditions. In addition, the ACL force was more influenced than the PCL force by the deficiency of PLC structures during the loading conditions of the gait cycle. The PT was the most influential PLC structure to provide significant effect on dynamic loading conditions. We also found that there was a remarkable increase in cruciate ligament force when the PLC structures such as LCL, PFL, and PT, were sectioned compared to the condition with PLC structures being intact.

Therefore, the first hypothesis was supported and the second hypothesis was rejected. Background knowledge about the importance of the individual anatomy of the posterolateral region of the knee joint for static stability was derived from sequential resections of these structures during static testing [7, 10, 11, 13]. However, it is challenging to assess intricate interactions and relationships regarding combinations of the different resections because of the correlations between the components of the PLC structure [12, 13]. Moreover, cadavers used during in vitro experiments are generally elderly humans; therefore, there may be not only loosening between the specimen and the testing device but also some weak tissue during loading conditions. In addition, most published in vitro studies, have considered only static loading and boundary conditions [5–10, 40].

A computational knee joint model allowed the exclusion of the disadvantages of an in vitro study, such as the inefficiency of cadaveric specimens during resection and repair, and limitations in static loading conditions. We introduced and validated and MSK knee model with 12-DOF (TF: 6-DOF, PF: 6-DOF) to simulate force-dependent kinematics during gait and squatting. The knee joint model was subjected to a series of rigorous validation steps, and the results showed good agreement with the results of an external rotation test and a previous experimental study [39]. There were prediction errors in the muscle activities calculated using the present computation model because of muscle redundancy under inverse dynamic conditions and inaccurate muscle moment arms (e.g., inaccurate muscle attachment points). However, unlike a previously reported study [17], the prediction results showed good agreement between the EMG measurements and muscle activities predicted by the computational model. In addition, the MSK models allows the representation of more realistic daily activities with muscle force interactions with, a wider range of knee flexions, whereas, current cadaver or finite element studies allow limited muscle activation [10–13, 18, 21, 41, 42].

No study has investigated the deficiency of PLC structures for both cruciate ligament forces under dynamic loading conditions. This is the first study that has evaluated both cruciate ligament forces following PLC resection under dynamic loading conditions. We also corrected popliteus muscle model to obtain a more faithful representation of its anatomy than the existing one. We found that the PLC structures, including the LCL, PFL, and PT, had a significant impact in the ACL and the PCL forces under dynamic conditions. Most previous studies have focused on the solely PLC and PCL [10, 33, 43–45]; however, the ACL is also a significant component of the knee joint, as shown by previous results [1, 2, 6–11]. It has been reported that 7.5–11% of all ACL injuries were accompanied by PLC structure injuries. However, few cadaveric static biomechanical studies have investigated the relationship between the ACL and PLC structures [46–48], and the proper biomechanical role of the ACL in PLC structure deficiency was not considered in previous studies [10, 43].

Bonanzinga et al. found that anterior-posterior (AP) laxity combined with a complete lesion of the PLC structure at 30° of knee flexion could be controlled in isolation, but not in hyperflexion of the knee joint [49]. At 90° of flexion, resection of the LCL led to a significant increase in terms of AP laxity and a significant difference in the internal-external (IE) laxity after PLC structure resection. This trend was also found in our study. Under particular stance phase gait-loading condition, a deficiency of all PLC structures and the LCL led to a greater increase in the force exerted on the ACL than on PCL. Conversely, deficiency of PLC structures led to a greater increase in the force exerted on the PCL than on the ACL when the flexion angle was high while squatting.

In this study, the PFL did not significantly affect the increased force exerted on the cruciate ligaments under gait-loading conditions. In previous studies, the PFL, rather than the popliteus muscle-tendon unit, was found to have a major role in external rotation stability [50, 51]. However, dynamic-loading conditions, rather than static-loading conditions, were used in this study, which may explain the contrasting results. In addition, studies have reported that the LCL had a greater influence with lower flexion- and mid-flexion angles in external rotation [10, 52]. Harner et al. stated that the popliteus muscle load decreased the posterior tibial translation by 2–3 mm, or by up to 36% of the increase in posterior tibial translation caused by PCL transection in a PCL-deficient knee model [53]. There is clinical speculation that, for patients with chronic PCL deficiency, PCL function could be replaced by a healthy popliteus muscle, thereby improving its function with specific physical exercises [53]. Höher et al reported that more force was exerted on the PCL than on the LCL with muscle activation of the PT during posterior drawer tests [54]. These results could evidence that the PFL did not significantly influence the cruciate ligaments under gait-loading conditions. In our MSK knee model, the effect of the PFL decreased due to the influences of other muscles. In other words, especially at low knee flexion angles, the role of the PFL became negligible and the PT undertook most of the mechanical work under dynamic gait loading conditions.

The importance of PT deficiency in the PLC structure was also suggested by a previous cadaveric study [13]. However, the PFL was more relevant under squat loading conditions, and its influence was not significant. Under squat loading conditions, deficiency of the PFL structure influenced the PCL more than the ACL. Under gait loading conditions, the most influential factor on the cruciate ligaments was the PT followed by the LCL and PFL. However, under squat loading conditions, the aPCL and pPCL were more influenced by the PFL than by the LCL. The present study showed that the effects of the LCL on the PCL decreased as the knee became highly flexed; these results were in agreement with those of previous static studies [12]. Both cruciate ligament forces markedly increased with deficiency of the total PLC structure, when compared to the selectively resected PLC structure, due to their synergetic effects. Under squat-loading conditions, the PCL force increased with the deficiency of the PLC structures, regardless of the flexion angle.

An important finding was that deficiency of the entire PLC structure caused greater PCL forces at high flexion angles, but greater ACL forces at low flexion angles, under squat loading conditions. The LCL significantly influenced the cruciate ligament forces at low knee flexion angles under gait loading conditions. PFL deficiency influenced only the pPCL bundle force under gait loading conditions, and the aPCL and pPCL bundle forces under squat loading conditions, but the results were not significant. The PT was the dynamic stabilizer and influenced the ACL and PCL forces under gait and squat loading conditions.

It is important to highlight several strengths of the present study. First, using one well-validated computational model is a proven method of evaluation of orthopedic biomechanics [15–21, 27–29, 35]. However, we did not use one well-validated computational model. Instead, we developed five different MSK models to statistically analyze the results. Second, we validated the results using strict criteria; results of experimental EMG, muscle activation from simulation, and external rotation from rotational test were compared with previous experimental data.

Nevertheless, several limitations of the present study should also be noted. First, we could not implement the deficiency of the PT alone because the PFL is connected to the PT in the model. Second, the ligaments were represented by only two or three bundles. Third, it is unclear how the force on the ligament, which was the outcome of interest of this study, could affect actual clinical outcomes. More research is needed to determine clinically meaningful detrimental force changes. Nevertheless, ligament forces are key factors that should be investigated to determine their roles the evaluation of biomechanical effects on computational biomechanics [12, 35, 45, 52, 55, 56]. Fourth, although our computational model was compared with EMG data and previous study, additional validation of ligament forces and parameters may be required. Finally, to improve bony structure wrapping, wrapped objects were included. However, these surfaces were modified into simple geometrical objects.

In terms of clinical relevance, posterolateral insufficiency of the knee affects significantly ACL and PCL forces in daily dynamic activity. Therefore PLC injuries associated with cruciate ligament injuries must be restored as well. In particular, surgical restoration or rehabilitation of the PT, which is the most crucial dynamic stabilizer, could be highlighted.

## Conclusions

The effect of the deficiency of each of the PLC structures on ACL and PCL forces was evaluated under dynamic loading conditions in this study. The deficiency of the entire PLC structures resulted in increased loadings on both PCL and ACL forces. The present results showed that PT is the most influential structure for the cruciate ligaments forces under dynamic loading conditions.

## Supporting information

S1 Fig (DOCX)

S1 Data (XLSX)

## References

[1] Fanelli GC. Posterior cruciate ligament injuries in trauma patients. Arthroscopy: the journal of arthroscopic & related surgery: official publication of the Arthroscopy Association of North America and the International Arthroscopy Association. 1993;9(3):291–4. Epub 1993/01/01. . 8323614 10.1016/s0749-8063(05)80424-4

[2] Fanelli GC, Edson CJ. Posterior cruciate ligament injuries in trauma patients: Part II. Arthroscopy: the journal of arthroscopic & related surgery: official publication of the Arthroscopy Association of North America and the International Arthroscopy Association. 1995;11(5):526–9. Epub 1995/10/01. . 8534292 10.1016/0749-8063(95)90127-2

[3] O'Brien SJ, Warren RF, Pavlov H, Panariello R, Wickiewicz TL. Reconstruction of the chronically insufficient anterior cruciate ligament with the central third of the patellar ligament. The Journal of bone and joint surgery American volume. 1991;73(2):278–86. Epub 1991/02/01. . 1993722

[4] Association AM. Standard nomenclature of athletic injuries: The Association; 1968.

[5] Maynard MJ, Deng X, Wickiewicz TL, Warren RF. The popliteofibular ligament. Rediscovery of a key element in posterolateral stability. Am J Sports Med. 1996;24(3):311–6. Epub 1996/05/01. 10.1177/036354659602400311 . 8734881

[6] Veltri DM, Deng XH, Torzilli PA, Warren RF, Maynard MJ. The role of the cruciate and posterolateral ligaments in stability of the knee. A biomechanical study. Am J Sports Med. 1995;23(4):436–43. Epub 1995/07/01. 10.1177/036354659502300411 . 7573653

[7] Veltri DM, Deng XH, Torzilli PA, Maynard MJ, Warren RF. The role of the popliteofibular ligament in stability of the human knee. A biomechanical study. Am J Sports Med. 1996;24(1):19–27. Epub 1996/01/01. 10.1177/036354659602400105 . 8638748

[8] Terry GC, LaPrade RF. The biceps femoris muscle complex at the knee. Its anatomy and injury patterns associated with acute anterolateral-anteromedial rotatory instability. Am J Sports Med. 1996;24(1):2–8. Epub 1996/01/01. 10.1177/036354659602400102 . 8638749

[9] Terry GC, LaPrade RF. The posterolateral aspect of the knee. Anatomy and surgical approach. Am J Sports Med. 1996;24(6):732–9. Epub 1996/11/01. 10.1177/036354659602400606 . 8947393

[10] Gollehon DL, Torzilli PA, Warren RF. The role of the posterolateral and cruciate ligaments in the stability of the human knee. A biomechanical study. The Journal of bone and joint surgery American volume. 1987;69(2):233–42. Epub 1987/02/01. . 3805084

[11] Grood ES, Stowers SF, Noyes FR. Limits of movement in the human knee. Effect of sectioning the posterior cruciate ligament and posterolateral structures. The Journal of bone and joint surgery American volume. 1988;70(1):88–97. Epub 1988/01/01. . 3335577

[12] LaPrade RF, Tso A, Wentorf FA. Force measurements on the fibular collateral ligament, popliteofibular ligament, and popliteus tendon to applied loads. Am J Sports Med. 2004;32(7):1695–701. Epub 2004/10/21. 10.1177/0363546503262694 . 15494335

[13] Chun YM, Kim SJ, Kim HS. Evaluation of the mechanical properties of posterolateral structures and supporting posterolateral instability of the knee. Journal of orthopaedic research: official publication of the Orthopaedic Research Society. 2008;26(10):1371–6. Epub 2008/04/12. 10.1002/jor.20596 . 18404705

[14] LaPrade RF, Muench C, Wentorf F, Lewis JL. The effect of injury to the posterolateral structures of the knee on force in a posterior cruciate ligament graft: a biomechanical study. Am J Sports Med. 2002;30(2):233–8. Epub 2002/03/26. 10.1177/03635465020300021501 . 11912094

[15] Dhaher YY, Kwon TH, Barry M. The effect of connective tissue material uncertainties on knee joint mechanics under isolated loading conditions. J Biomech. 2010;43(16):3118–25. 10.1016/j.jbiomech.2010.08.005 20810114 PMC3641768

[16] Halloran JP, Ackermann M, Erdemir A, van den Bogert AJ. Concurrent musculoskeletal dynamics and finite element analysis predicts altered gait patterns to reduce foot tissue loading. J Biomech. 2010;43(14):2810–5. Epub 2010/06/25. 10.1016/j.jbiomech.2010.05.036 20573349 PMC2946980

[17] Chen Z, Zhang X, Ardestani MM, Wang L, Liu Y, Lian Q, et al Prediction of in vivo joint mechanics of an artificial knee implant using rigid multi-body dynamics with elastic contacts. Proceedings of the Institution of Mechanical Engineers Part H, Journal of engineering in medicine. 2014;228(6):564–75. Epub 2014/06/01. 10.1177/0954411914537476 . 24878735

[18] Lenhart RL, Kaiser J, Smith CR, Thelen DG. Prediction and validation of load-dependent behavior of the tibiofemoral and patellofemoral joints during movement. Annals of biomedical engineering. 2015;43(11):2675–85. Epub 2015/04/29. 10.1007/s10439-015-1326-3 . 25917122 PMC4886716

[19] Chen Z, Wang L, Liu Y, He J, Lian Q, Li D, et al Effect of component mal-rotation on knee loading in total knee arthroplasty using multi-body dynamics modeling under a simulated walking gait. Journal of orthopaedic research: official publication of the Orthopaedic Research Society. 2015;33(9):1287–96. Epub 2015/03/31. 10.1002/jor.22908 . 25820991

[20] Shelburne KB, Torry MR, Pandy MG. Contributions of muscles, ligaments, and the ground-reaction force to tibiofemoral joint loading during normal gait. Journal of orthopaedic research: official publication of the Orthopaedic Research Society. 2006;24(10):1983–90. Epub 2006/08/11. 10.1002/jor.20255 . 16900540

[21] Shin CS, Chaudhari AM, Andriacchi TP. The influence of deceleration forces on ACL strain during single-leg landing: a simulation study. J Biomech. 2007;40(5):1145–52. Epub 2006/06/27. 10.1016/j.jbiomech.2006.05.004 . 16797556

[22] Thelen DG, Won Choi K, Schmitz AM. Co-simulation of neuromuscular dynamics and knee mechanics during human walking. Journal of biomechanical engineering. 2014;136(2):021033 Epub 2014/01/07. 10.1115/1.4026358 24390129 PMC4023657

[23] Lloyd DG, Besier TF. An EMG-driven musculoskeletal model to estimate muscle forces and knee joint moments in vivo. J Biomech. 2003;36(6):765–76. Epub 2003/05/14. . 12742444 10.1016/s0021-9290(03)00010-1

[24] Klein Horsman MD, Koopman HF, van der Helm FC, Prose LP, Veeger HE. Morphological muscle and joint parameters for musculoskeletal modelling of the lower extremity. Clinical biomechanics. 2007;22(2):239–47. Epub 2006/12/01. 10.1016/j.clinbiomech.2006.10.003 . 17134801

[25] Forster E. Predicting muscle forces in the human lower limb during locomotion. Ulm University 2004.

[26] Ali N, Andersen MS, Rasmussen J, Robertson DG, Rouhi G. The application of musculoskeletal modeling to investigate gender bias in non-contact ACL injury rate during single-leg landings. Computer methods in biomechanics and biomedical engineering. 2014;17(14):1602–16. Epub 2013/02/08. 10.1080/10255842.2012.758718 . 23387967

[27] Kwon OR, Kang KT, Son J, Kwon SK, Jo SB, Suh DS, et al Biomechanical comparison of fixed- and mobile-bearing for unicomparmental knee arthroplasty using finite element analysis. Journal of orthopaedic research: official publication of the Orthopaedic Research Society. 2014;32(2):338–45. Epub 2013/10/15. 10.1002/jor.22499 . 24122942

[28] Kim YS, Kang KT, Son J, Kwon OR, Choi YJ, Jo SB, et al Graft Extrusion Related to the Position of Allograft in Lateral Meniscal Allograft Transplantation: Biomechanical Comparison Between Parapatellar and Transpatellar Approaches Using Finite Element Analysis. Arthroscopy: the journal of arthroscopic & related surgery: official publication of the Arthroscopy Association of North America and the International Arthroscopy Association. 2015;31(12):2380–91.e2. Epub 2015/09/08. 10.1016/j.arthro.2015.06.030 . 26343943

[29] Marra MA, Vanheule V, Fluit R, Koopman BH, Rasmussen J, Verdonschot N, et al A subject-specific musculoskeletal modeling framework to predict in vivo mechanics of total knee arthroplasty. Journal of biomechanical engineering. 2015;137(2):020904 Epub 2014/11/28. 10.1115/1.4029258 . 25429519

[30] Bowman KF Jr., Sekiya JK. Anatomy and biomechanics of the posterior cruciate ligament, medial and lateral sides of the knee. Sports medicine and arthroscopy review. 2010;18(4):222–9. Epub 2010/11/17. 10.1097/JSA.0b013e3181f917e2 . 21079500

[31] Baldwin JL. The anatomy of the medial patellofemoral ligament. The American journal of sports medicine. 2009;37(12):2355–61. Epub 2009/09/05. 10.1177/0363546509339909 . 19729366

[32] Amis AA, Firer P, Mountney J, Senavongse W, Thomas NP. Anatomy and biomechanics of the medial patellofemoral ligament. Knee. 2003;10(3):215–20. Epub 2003/08/02. . 12893142 10.1016/s0968-0160(03)00006-1

[33] Kang KT, Koh YG, Son J, Jung M, Oh S, Kim SJ, et al Biomechanical influence of deficient posterolateral corner structures on knee joint kinematics: A computational study. Journal of orthopaedic research: official publication of the Orthopaedic Research Society. 2018 Epub 2018/02/14. 10.1002/jor.23871 . 29436742

[34] Blankevoort L, Huiskes R. Ligament-bone interaction in a three-dimensional model of the knee. Journal of biomechanical engineering. 1991;113(3):263–9. Epub 1991/08/01. . 1921352 10.1115/1.2894883

[35] Li G, Gil J, Kanamori A, Woo SL. A validated three-dimensional computational model of a human knee joint. Journal of biomechanical engineering. 1999;121(6):657–62. Epub 2000/01/14. . 10633268 10.1115/1.2800871

[36] Fregly BJ, Bei Y, Sylvester ME. Experimental evaluation of an elastic foundation model to predict contact pressures in knee replacements. J Biomech. 2003;36(11):1659–68. Epub 2003/10/03. . 14522207 10.1016/s0021-9290(03)00176-3

[37] LaPrade RF, Moulton SG, Nitri M, Mueller W, Engebretsen L. Clinically relevant anatomy and what anatomic reconstruction means. Knee surgery, sports traumatology, arthroscopy: official journal of the ESSKA. 2015;23(10):2950–9. Epub 2015/05/11. 10.1007/s00167-015-3629-1 . 25957611

[38] Skipper Andersen M, de Zee M, Damsgaard M, Nolte D, Rasmussen J. Introduction to Force-Dependent Kinematics: Theory and Application to Mandible Modeling. Journal of biomechanical engineering. 2017;139(9). Epub 2017/06/24. 10.1115/1.4037100 . 28639682

[39] McCarthy M, Camarda L, Wijdicks CA, Johansen S, Engebretsen L, Laprade RF. Anatomic posterolateral knee reconstructions require a popliteofibular ligament reconstruction through a tibial tunnel. Am J Sports Med. 2010;38(8):1674–81. Epub 2010/08/03. 10.1177/0363546510361220 . 20675651

[40] Ullrich K, Krudwig WK, Witzel U. Posterolateral aspect and stability of the knee joint. I. Anatomy and function of the popliteus muscle-tendon unit: an anatomical and biomechanical study. Knee surgery, sports traumatology, arthroscopy: official journal of the ESSKA. 2002;10(2):86–90. Epub 2002/03/27. 10.1007/s00167-001-0268-5 . 11914765

[41] Liau JJ, Cheng CK, Huang CH, Lo WH. The effect of malalignment on stresses in polyethylene component of total knee prostheses—a finite element analysis. Clinical biomechanics (Bristol, Avon). 2002;17(2):140–6. Epub 2002/02/08. . 11832264 10.1016/s0268-0033(01)00109-7

[42] Colwell CW Jr., Chen PC, D'Lima D. Extensor malalignment arising from femoral component malrotation in knee arthroplasty: effect of rotating-bearing. Clinical biomechanics. 2011;26(1):52–7. Epub 2010/09/28. 10.1016/j.clinbiomech.2010.08.009 . 20869142

[43] Zantop T, Schumacher T, Diermann N, Schanz S, Raschke MJ, Petersen W. Anterolateral rotational knee instability: role of posterolateral structures. Winner of the AGA-DonJoy Award 2006. Archives of orthopaedic and trauma surgery. 2007;127(9):743–52. Epub 2006/10/31. 10.1007/s00402-006-0241-3 . 17072626

[44] Vogrin TM, Hoher J, Aroen A, Woo SL, Harner CD. Effects of sectioning the posterolateral structures on knee kinematics and in situ forces in the posterior cruciate ligament. Knee surgery, sports traumatology, arthroscopy: official journal of the ESSKA. 2000;8(2):93–8. Epub 2000/05/05. 10.1007/s001670050193 . 10795671

[45] Kang KT, Koh YG, Jung M, Nam JH, Son J, Lee YH, et al The effects of posterior cruciate ligament deficiency on posterolateral corner structures under gait- and squat-loading conditions: A computational knee model. Bone & joint research. 2017;6(1):31–42. Epub 2017/01/13. 10.1302/2046-3758.61.bjr-2016-0184.r1 . 28077395 PMC5301905

[46] Kim SJ, Choi DH, Hwang BY. The influence of posterolateral rotatory instability on ACL reconstruction: comparison between isolated ACL reconstruction and ACL reconstruction combined with posterolateral corner reconstruction. The Journal of bone and joint surgery American volume. 2012;94(3):253–9. Epub 2012/02/03. 10.2106/JBJS.J.01686 . 22298058

[47] Fanelli GC, Larson RV. Practical management of posterolateral instability of the knee. Arthroscopy: the journal of arthroscopic & related surgery: official publication of the Arthroscopy Association of North America and the International Arthroscopy Association. 2002;18(2 Suppl 1):1–8. Epub 2002/02/06. . 11828342 10.1053/jars.2002.31779

[48] Ross G, DeConciliis GP, Choi K, Scheller AD. Evaluation and treatment of acute posterolateral corner/anterior cruciate ligament injuries of the knee. The Journal of bone and joint surgery American volume. 2004;86-A Suppl 2:2–7. Epub 2005/02/05. . 15691102 10.2106/00004623-200412002-00002

[49] Bonanzinga T, Signorelli C, Lopomo N, Grassi A, Neri MP, Filardo G, et al Biomechanical effect of posterolateral corner sectioning after ACL injury and reconstruction. Knee surgery, sports traumatology, arthroscopy: official journal of the ESSKA. 2015;23(10):2918–24. Epub 2015/07/18. 10.1007/s00167-015-3696-3 . 26183733

[50] Shahane SA, Ibbotson C, Strachan R, Bickerstaff DR. The popliteofibular ligament. An anatomical study of the posterolateral corner of the knee. The Journal of bone and joint surgery British volume. 1999;81(4):636–42. Epub 1999/08/27. . 10463736 10.1302/0301-620x.81b4.9501

[51] Sugita T, Amis AA. Anatomic and biomechanical study of the lateral collateral and popliteofibular ligaments. Am J Sports Med. 2001;29(4):466–72. Epub 2001/07/31. 10.1177/03635465010290041501 . 11476388

[52] LaPrade RF, Resig S, Wentorf F, Lewis JL. The effects of grade III posterolateral knee complex injuries on anterior cruciate ligament graft force. A biomechanical analysis. Am J Sports Med. 1999;27(4):469–75. Epub 1999/07/29. 10.1177/03635465990270041101 . 10424217

[53] Harner CD, Hoher J, Vogrin TM, Carlin GJ, Woo SL. The effects of a popliteus muscle load on in situ forces in the posterior cruciate ligament and on knee kinematics. A human cadaveric study. Am J Sports Med. 1998;26(5):669–73. Epub 1998/10/24. 10.1177/03635465980260051201 . 9784814

[54] Hoher J, Harner CD, Vogrin TM, Baek GH, Carlin GJ, Woo SL. In situ forces in the posterolateral structures of the knee under posterior tibial loading in the intact and posterior cruciate ligament-deficient knee. Journal of orthopaedic research: official publication of the Orthopaedic Research Society. 1998;16(6):675–81. Epub 1999/01/07. 10.1002/jor.1100160608 . 9877391

[55] Kang KT, Koh YG, Son J, Kwon OR, Baek C, Jung SH, et al Measuring the effect of femoral malrotation on knee joint biomechanics for total knee arthroplasty using computational simulation. Bone & joint research. 2016;5(11):552–9. Epub 2017/01/18. 10.1302/2046-3758.511.bjr-2016-0107.r1 28094763 PMC5131092

[56] Thompson JA, Hast MW, Granger JF, Piazza SJ, Siston RA. Biomechanical effects of total knee arthroplasty component malrotation: a computational simulation. Journal of orthopaedic research: official publication of the Orthopaedic Research Society. 2011;29(7):969–75. Epub 2011/05/14. 10.1002/jor.21344 . 21567450

