# Supplementary material for: Biomechanical evaluation of the influence of posterolateral corner structures on cruciate ligaments forces during simulated gait and squatting
Source: PLoS One. 2019 Apr 4;14(4):e0214496. doi: 10.1371/journal.pone.0214496 (PMC6448852; doi:10.1371/journal.pone.0214496)

**Appendix**

**S1 Fig.** The comparison between measured and predicted muscle activations with 5 subject-specific musculoskeletal models under (a) normal gait and (b) squat loading conditions.


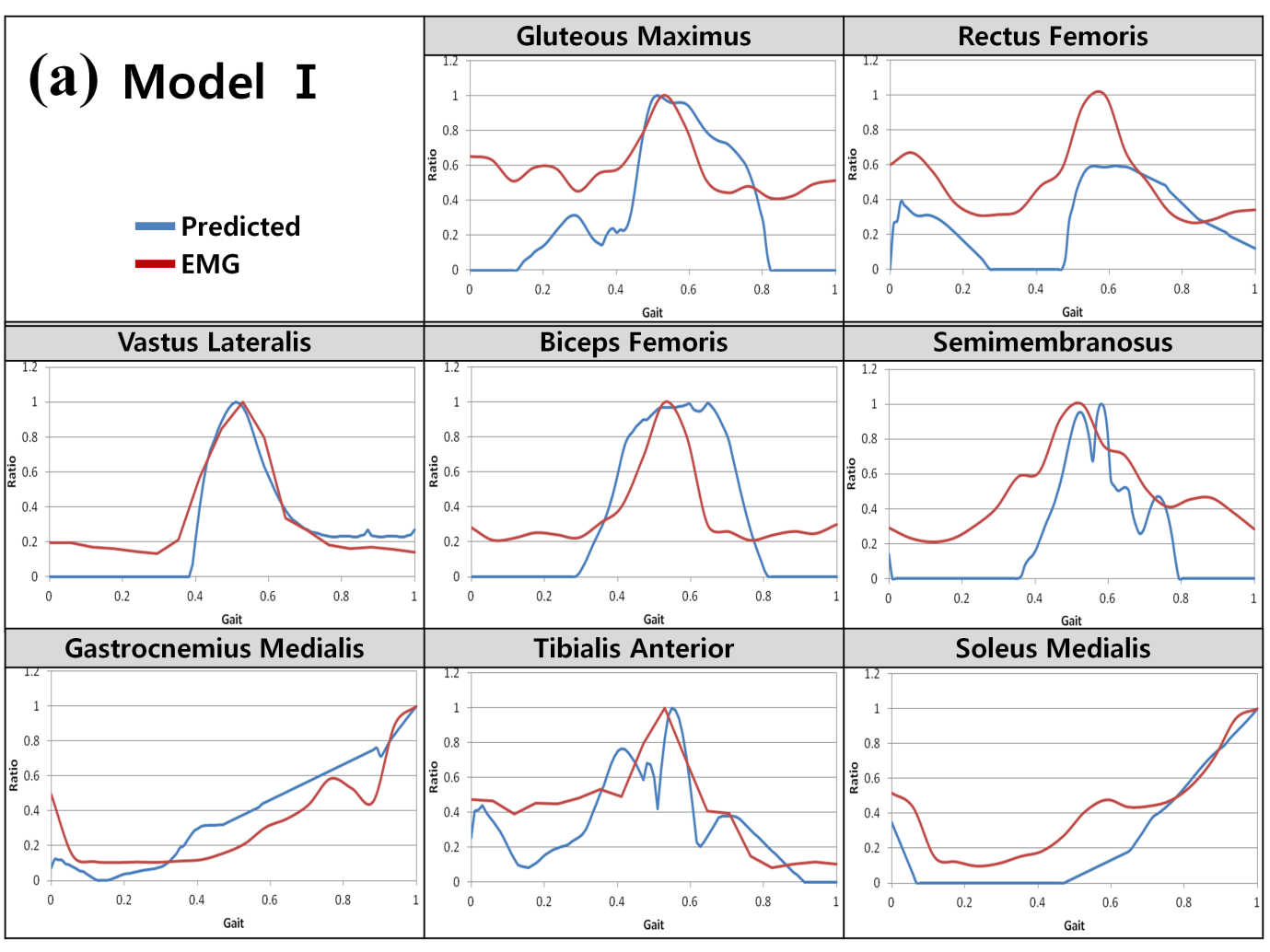


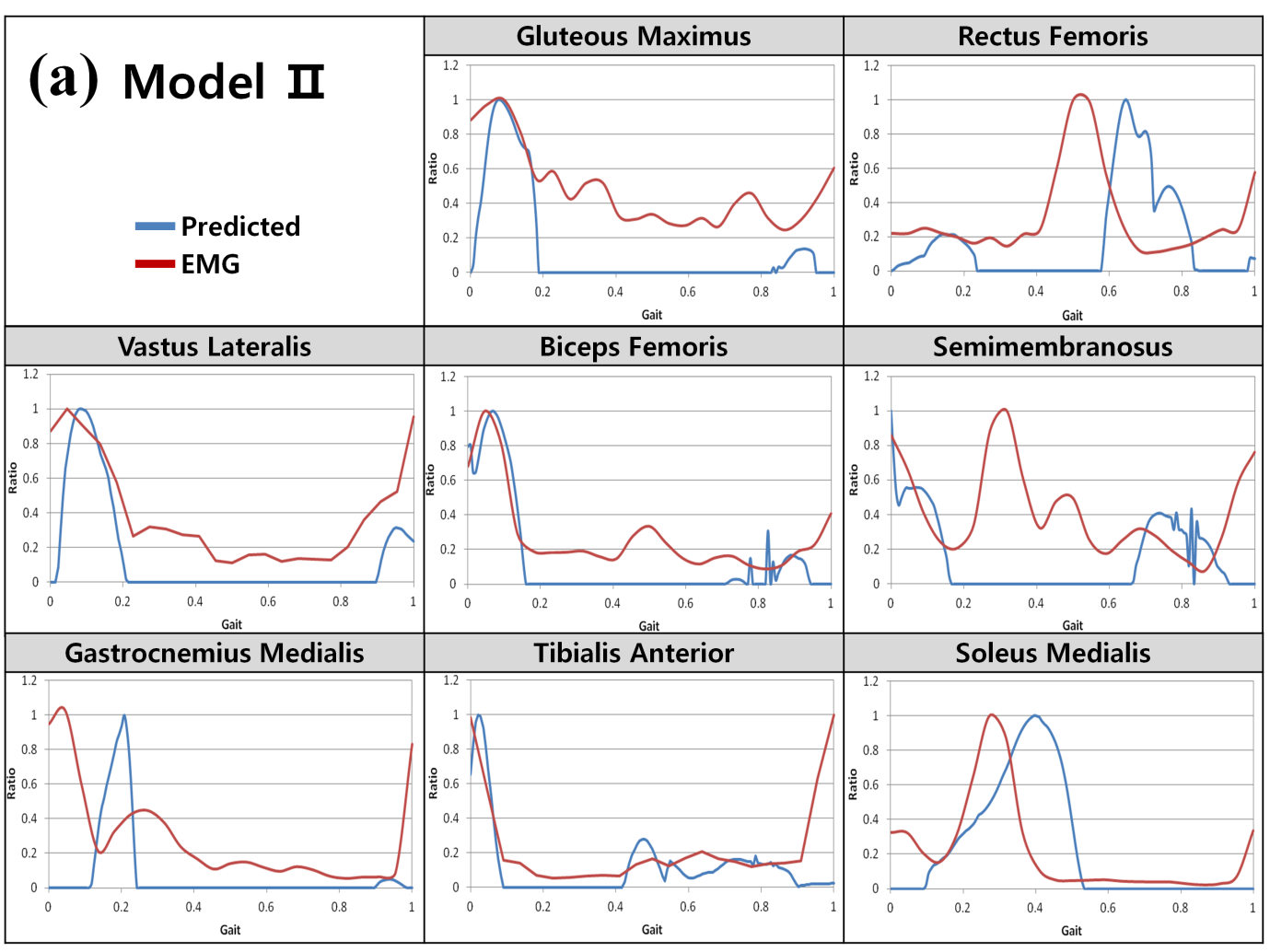


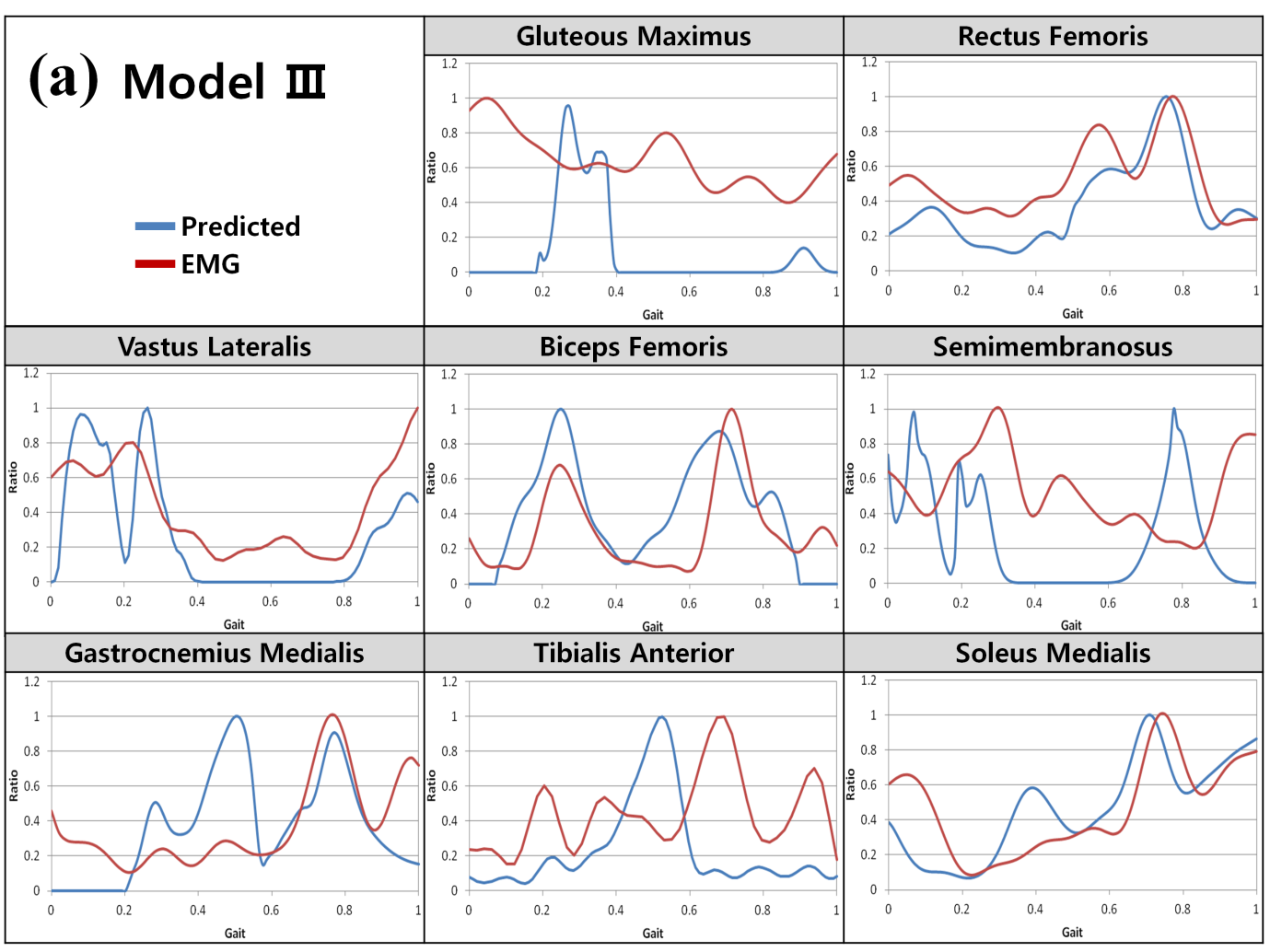


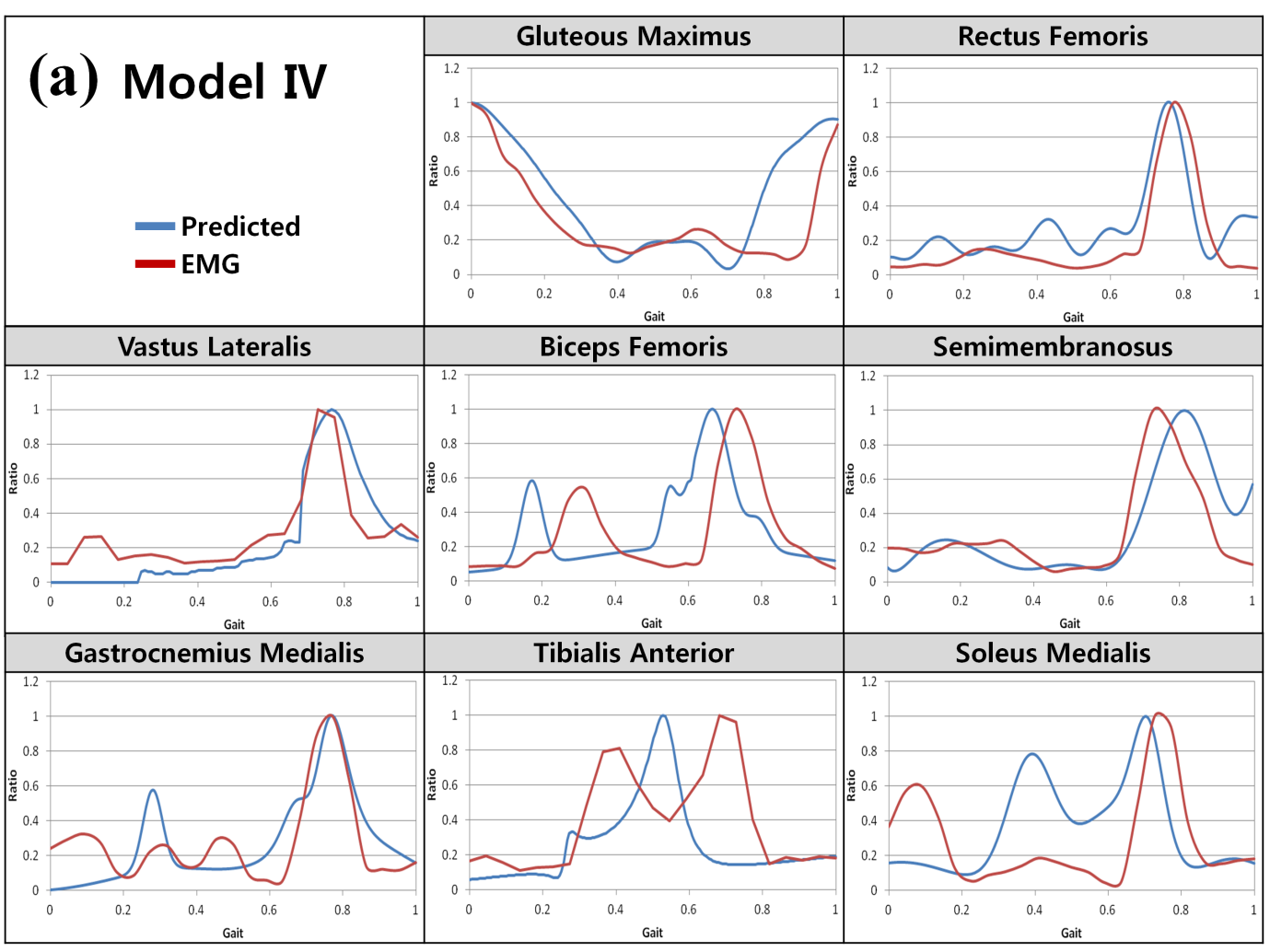


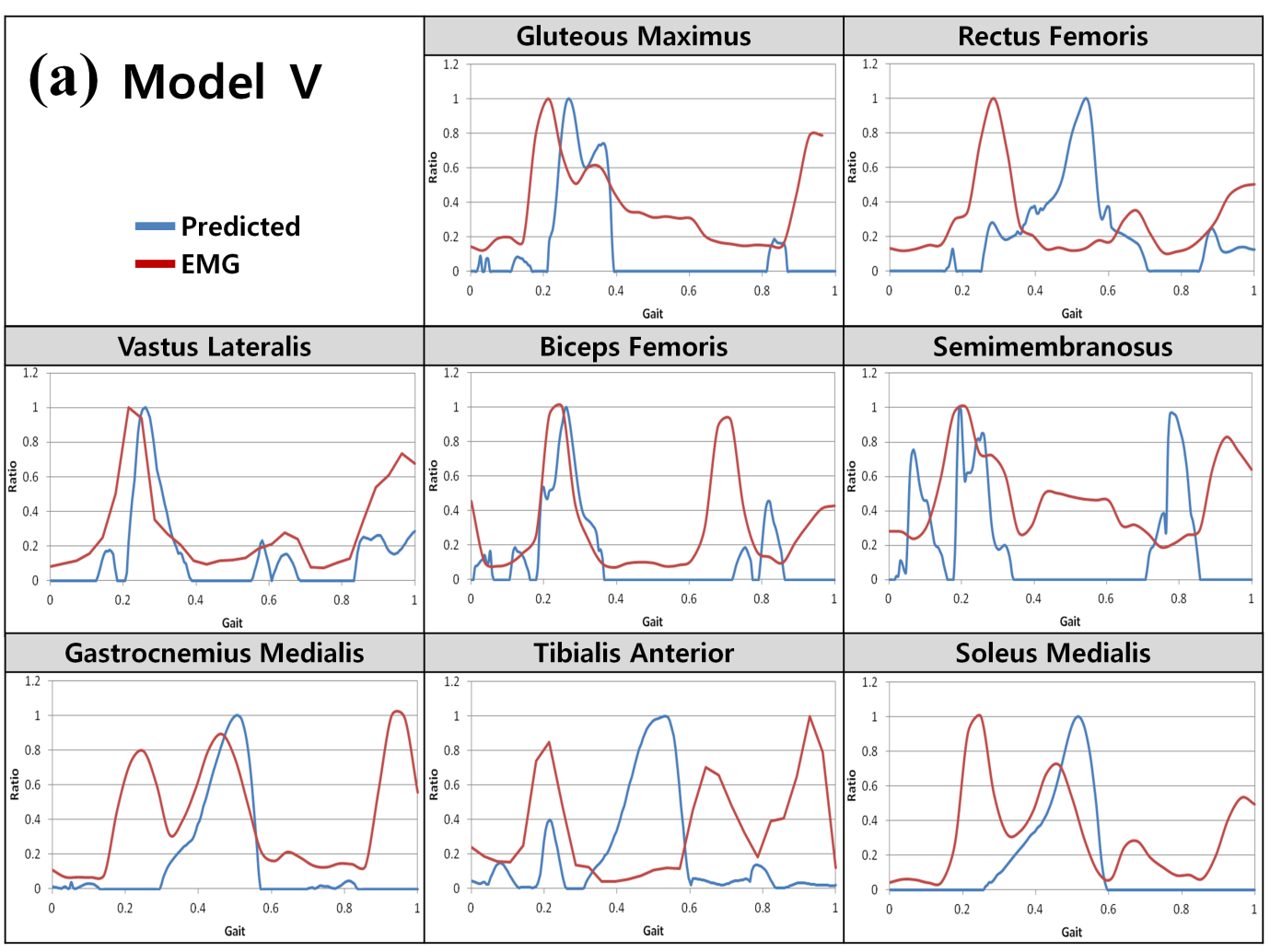


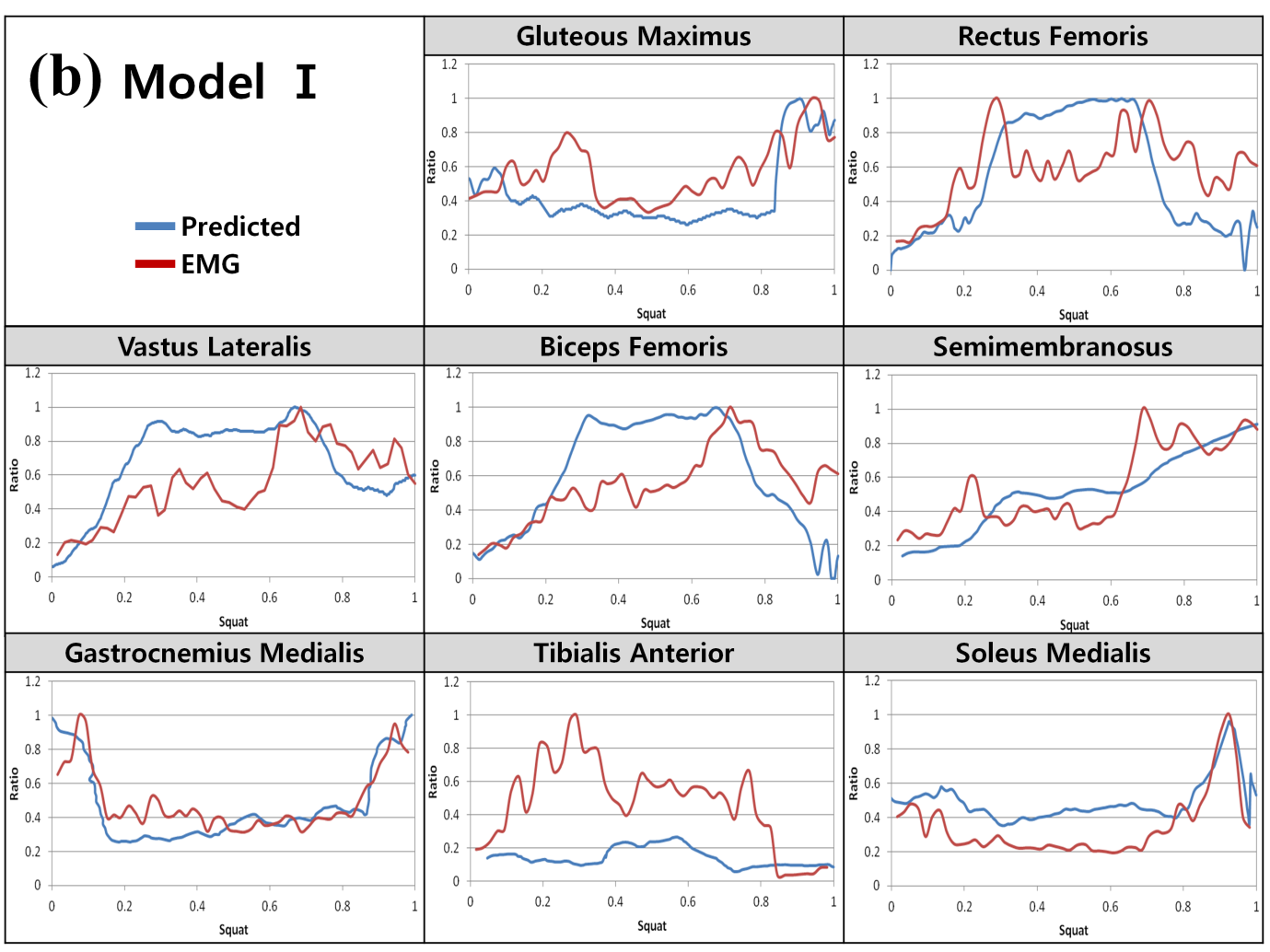


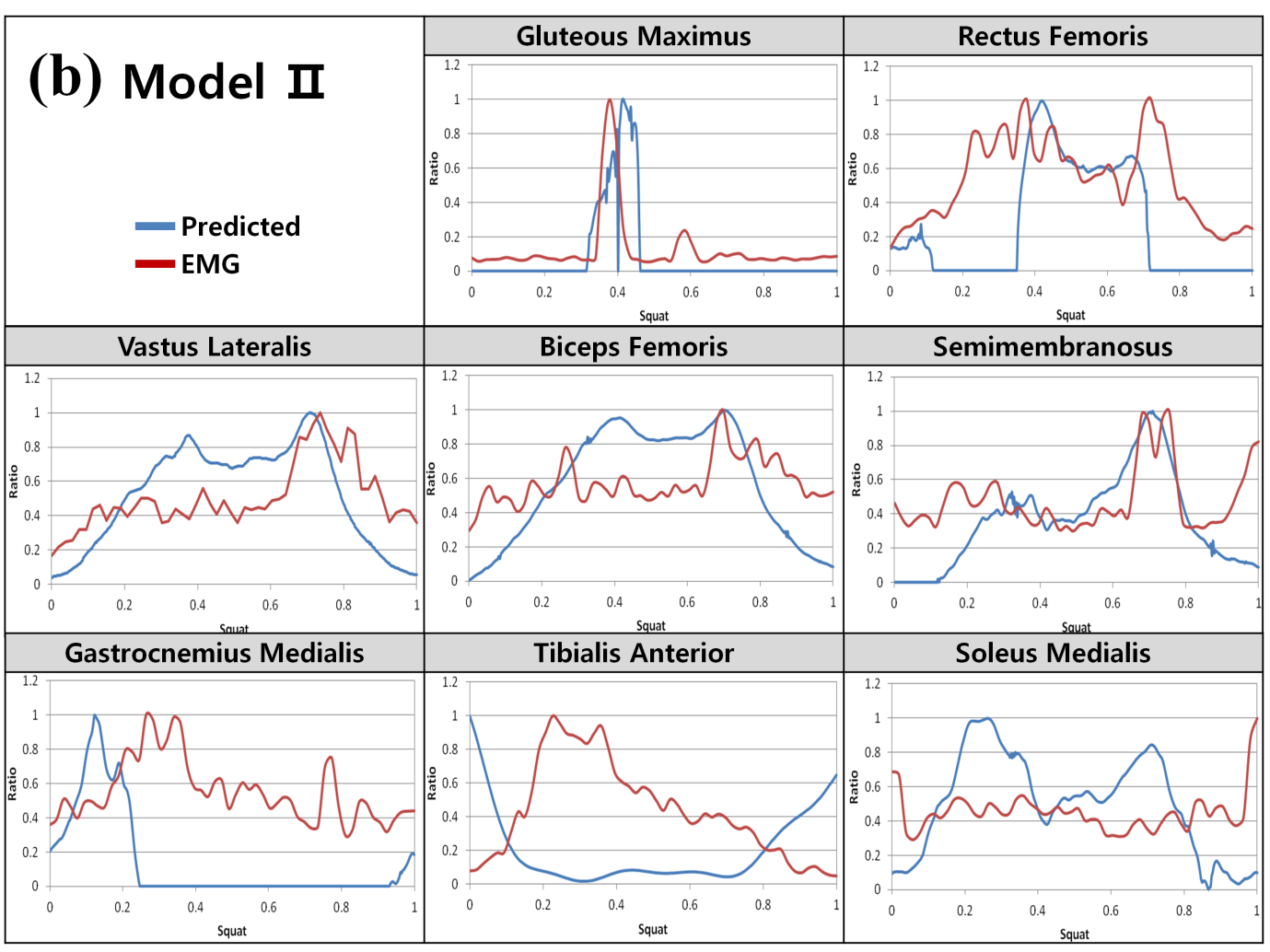


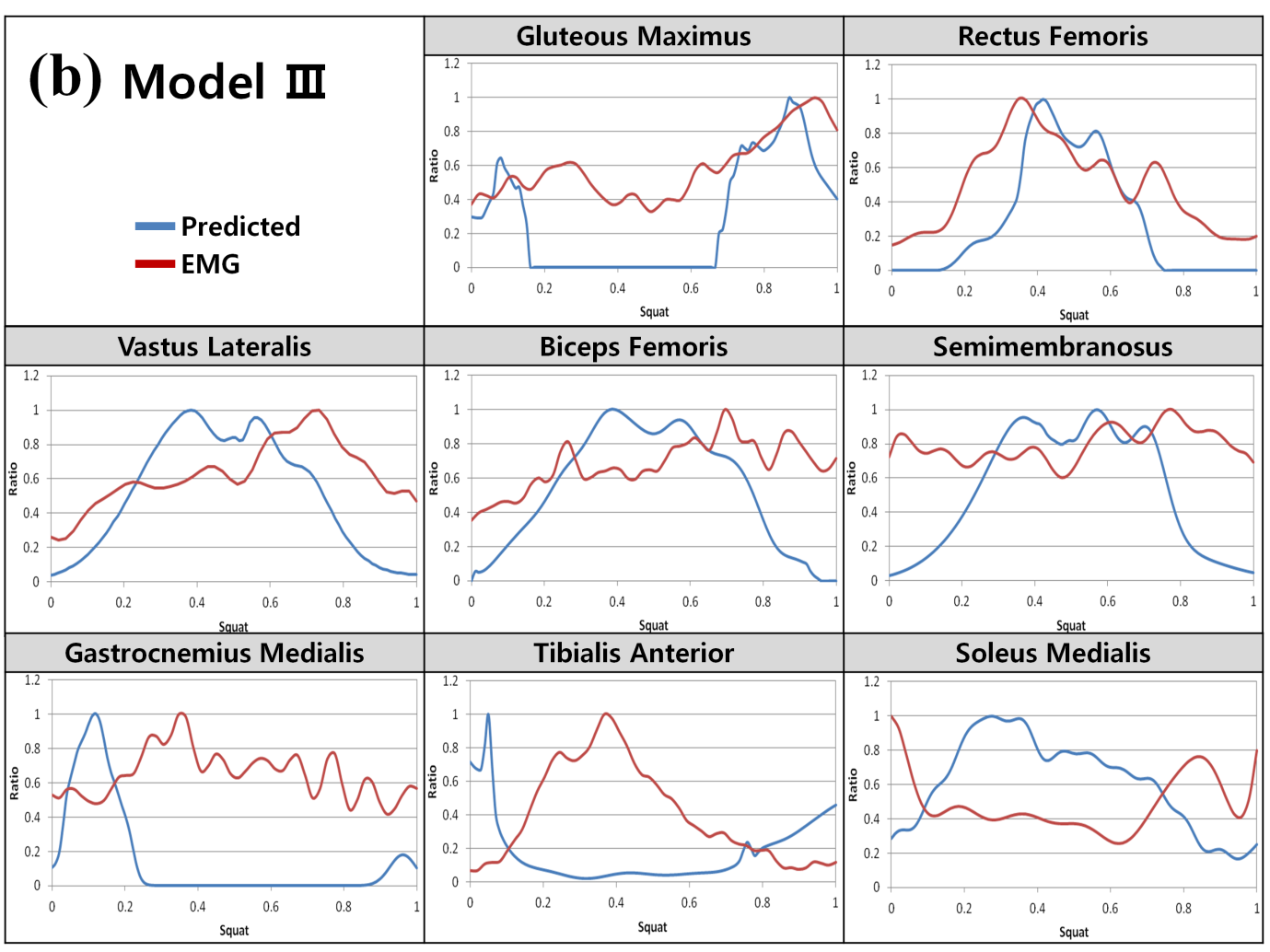


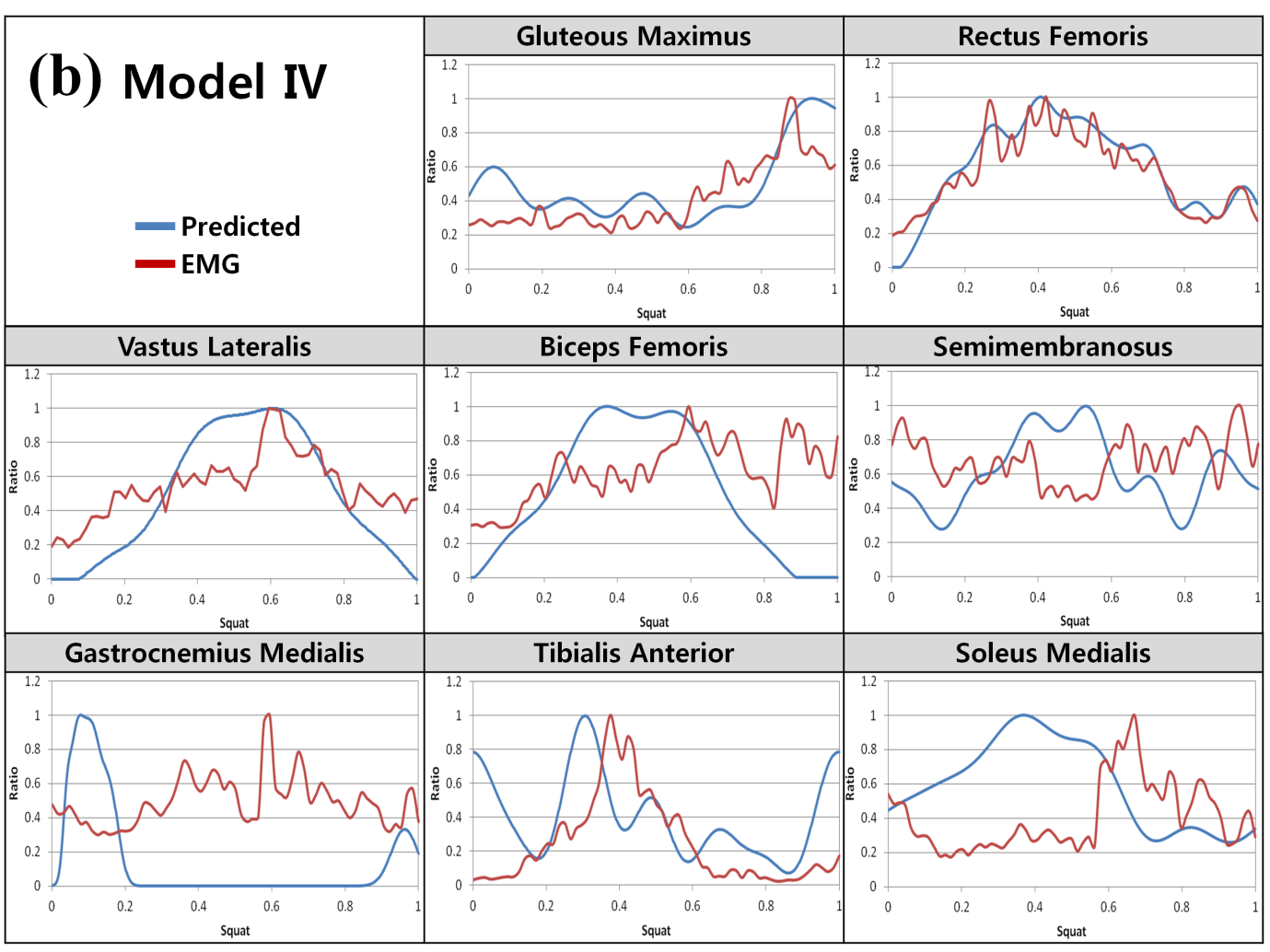


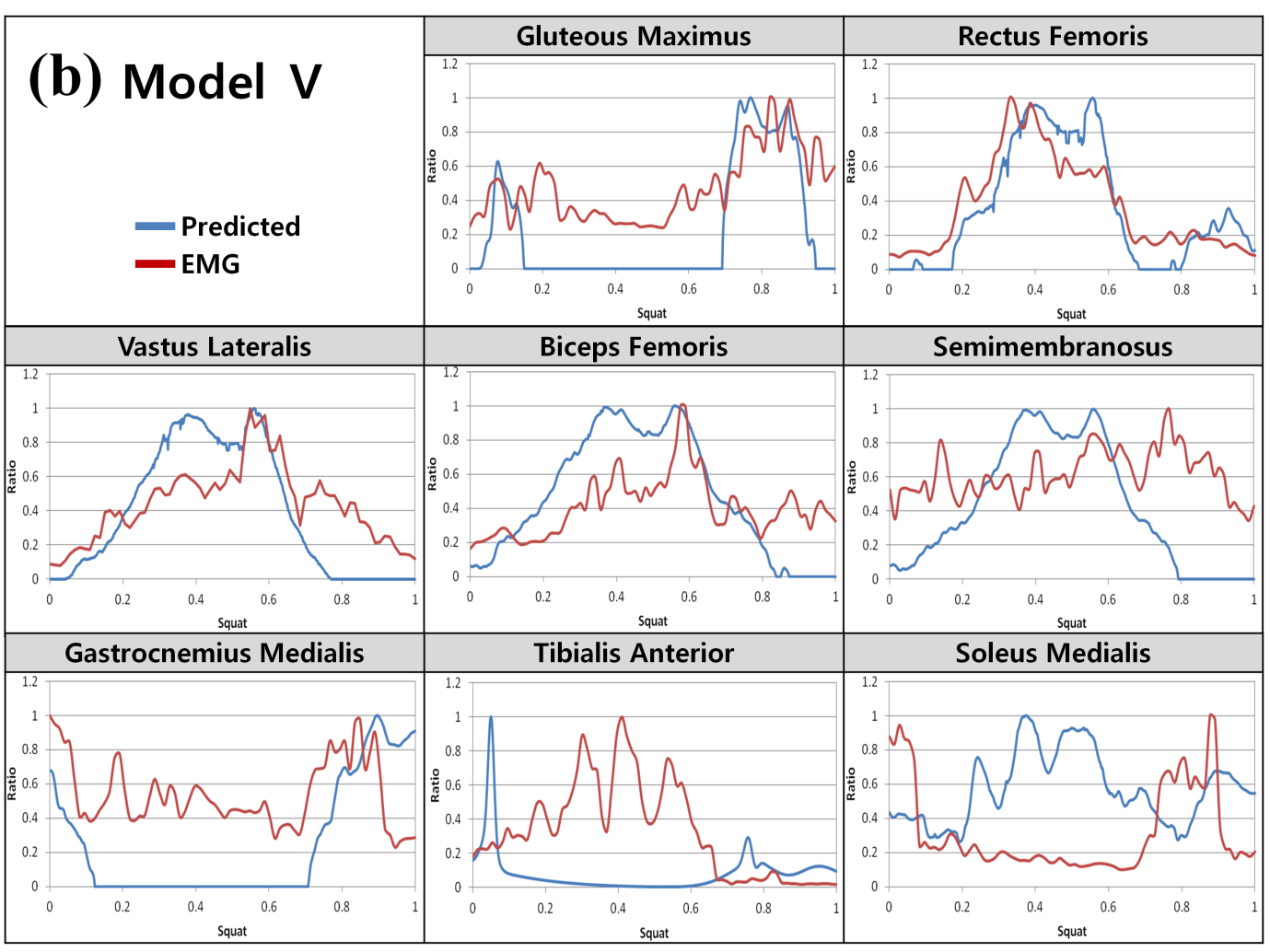

Supplement: S1 Fig — (DOCX) [file pone.0214496.s001.docx]
